# Measurement of COVID-19 related mental health problems

**DOI:** 10.1192/j.eurpsy.2022.1302

**Published:** 2022-09-01

**Authors:** M. Arts, S. Petrykiv, L. De Jonge

**Affiliations:** 1 GGZWNB, Psychiatry, Halsteren, Netherlands; 2 Leonardo Scientific Research Institute, Neuropsychiatry, Halsteren, Netherlands

**Keywords:** mental health, Covid-19

## Abstract

**Introduction:**

The spread of the corona virus (COVID-19) has an enormous psychosocial impact on humanity across the globe, resulting in an increase in mental health issues. There are no specific diagnostic instruments that could identify COVID-19 related mental health problems. In recent months, new scales have been developed to identify COVID-19 related problems.

**Objectives:**

Our objective was to investigate the clinical utility of these new assessment instruments.

**Methods:**

We performed a literature search, using Pubmed, EMBASE, Scopus and Cochrane library databases, to search for new scales identifying COVID-19 related mental health problems.

**Results:**

During the first half of the year 2020, we found five published new self-report measurement instruments: Coronavirus Anxiety Scale (CAS), the COVID Stress Scales (CSS), the Fear of COVID-19 Scale (FCV-19S), the Obsession with COVID-19 Scale (OCS), and the Questionnaire on Perception of Threat from COVID-19. These instruments have been validated in a group of middle-aged ambulatory patients.

**Conclusions:**

These new instruments might be useful in non-clinical settings. Although the psychometric reports are promising, the instruments have been validated in a less vulnerable group of patients. Future validation studies should also comprise other age groups, particularly the old and more vulnerable population.

**Disclosure:**

No significant relationships.

